# Development and evaluation of a computerised clinical decision support system for switching drugs at the interface between primary and tertiary care

**DOI:** 10.1186/1472-6947-12-137

**Published:** 2012-11-27

**Authors:** Markus G Pruszydlo, Stefanie U Walk-Fritz, Torsten Hoppe-Tichy, Jens Kaltschmidt, Walter E Haefeli

**Affiliations:** 1 Department of Clinical Pharmacology and Pharmacoepidemiology, University of Heidelberg, Heidelberg, Germany; 2 Hospital Pharmacy, University of Heidelberg, Heidelberg, Germany

**Keywords:** Clinical decision support systems, Drug information services, Drug switching

## Abstract

**Background:**

Upon admission to a hospital patients’ medications are frequently switched to alternative drugs compiled in so called hospital drug formularies. This substitution process is a laborious and error-prone task which should be supported by sophisticated electronic tools. We developed a computerised decision support system and evaluated benefit and potential harm associated with its use.

**Methods:**

Based on a multi-step algorithm we identified drug classes suitable for exchange, defined conversion factors for therapeutic interchange, built a web-based decision support system, and implemented it into the computerised physician order entry of a large university hospital. For evaluation we compared medications manually switched by clinical pharmacists with the results of automated switching by the newly developed computer system and optimised the system in an iterative process. Thereafter the final system was tested in an independent set of prescriptions.

**Results:**

After iterative optimisation of the logical framework the tool was able to switch drugs to pharmaceutical equivalents and alternatives; in addition, it contained 21 different drug classes for therapeutic substitution. In this final version it switched 91.6% of 202 documented medication consultations (containing 1,333 drugs) automatically, leaving 8.4% for manual processing by clinical professionals. No incorrect drug switches were found.

**Conclusion:**

A large majority (>90%) of drug switches performed at the interface between primary and tertiary care can be handled automatically using electronic decision support systems, indicating that medication errors and workload of healthcare professionals can be considerably reduced.

## Background

Drug switching at the interface between primary and tertiary care is a time-consuming and error-prone task in inpatient care [1]. In hospitals local drug committees and pharmacies usually define hospital drug formularies (HDF). HDF are restricted prescribing lists containing a subset of all available drugs with the intention to assure quality of in-house prescribing, simplify logistics, and save costs in drug therapy [2,3]. Given the large size of the German drug market the likelihood of drugs being on the HDF is small. Indeed, upon admission to a German hospital about 50% of all previous drugs are switched [1,4]. Appropriate switching is a laborious task that must carefully consider combination products, routes of administration, and also therapeutic equivalents and their (often differing) doses, if the same active ingredient is not available.

In a pilot investigation assessing the quality of drug switching we evaluated 128 switches in 30 consecutively admitted patients and learnt that one in five drug substitutions (21%) was wrong, mainly due to dose errors or mistakes in switching combination drugs to multiple single agents. The number and severity of these errors, which compromised optimum treatment already on the first day of hospitalisation, was alarming and we, thus, decided to support the process of drug switching electronically.

Computerised physician order entry (CPOE) and clinical decision support systems (CDSS) have successfully prevented prescription errors, improved patient outcome, and reduced cost [5-9].

To implement a CDSS for drug switching upon admission we developed a multi-step algorithm and in a first evaluation it qualified as a logical framework to ease the practice of switching drugs in a standardised and reliable way [1]. We then built a CDSS, based on the algorithm, implemented it into the CPOE of a large university hospital, and evaluated benefit and potential harm.

## Methods

### Development and implementation of the CDSS

**Figure 1:**
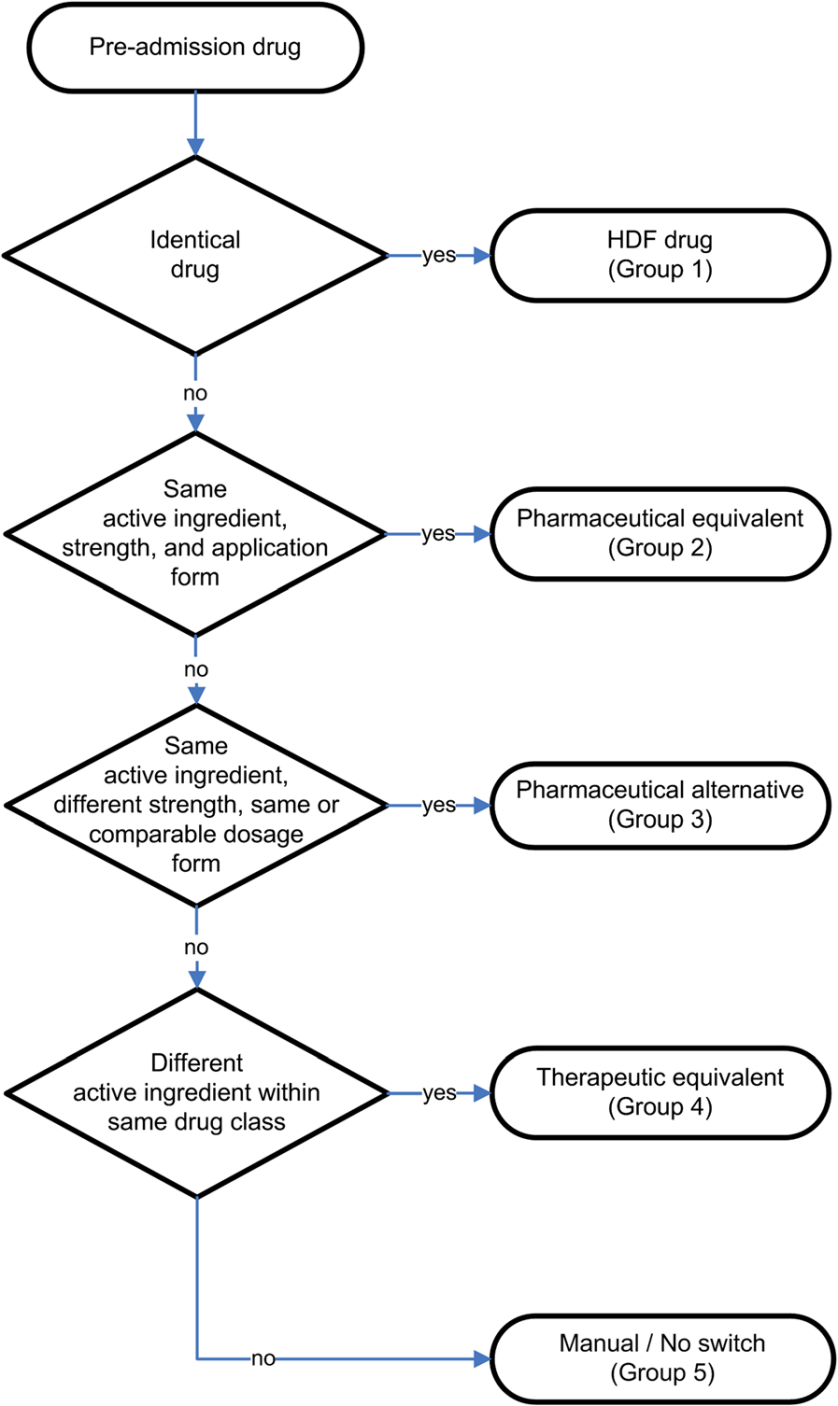
**Switch Algorithm.** Standardised multi-step algorithm to translate a drug regimen into appropriate alternatives contained in a hospital formulary (modified from [1]).

**Figure 2:**
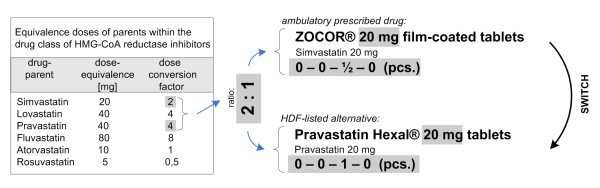
**Substitution of therapeutic equivalents.** Switch of a drug to a therapeutic equivalent within the same drug class and corresponding dose adjustment using dose conversion factors.

The logical basis of our CDSS was a multi-step algorithm that translates the drugs of a patient’s drug history into a suitable alternative therapy [1] (Figure 1) that was developed by an interdisciplinary team of specialists (physicians, pharmacists, and computer scientists). Briefly, in the first step the patients’ drug list is checked for drugs listed on the HDF. In this case no switch is necessary and the same drug is recommended. In a second step the HDF is searched for *pharmaceutical equivalents*, i.e. drugs with matching active ingredient (drug parent), strength, and application form. If no pharmaceutical equivalents are available, the algorithm seeks for *pharmaceutical alternatives*, i.e. drugs on the HDF with the same drug parent but differing strength and comparable dosage forms. Because of differing drug strengths the CDSS must be able to modify the dosage regimen adequately. If no pharmaceutical alternatives are identified for a drug the HDF is checked for *therapeutic equivalents* (Figure 2), defined as exchangeable drugs with differing parents within the same drug class.

Drugs within a drug-class can differ widely in potency and efficacy [10,11], which has to be considered during exchange to avoid over- and underdosing. Therefore, 21 drug classes were defined eligible for automatic interchange and corresponding dose conversion factors were extracted from the literature (Table 1). If no head-to-head comparison of a switch pair was published, conversion factors were derived from the approved maintenance doses as published in the summary of product characteristics (SPC). These classes usually contained drugs of a common Anatomic Therapeutic Chemical (ATC) classification system group (e.g. calcium-channel blocking agents; Table 1) and concurrently considered approved medical indications of the compound to be switched. Hence, if calcium channel blockers were to be switched, the system did not suggest the substitution of felodipine (indication: hypertension) with nimodipine (indication: prevention of cerebral vasospasm) because of the differing labelled indications. If a drug was approved for more than one indication (e.g. ramipril for hypertension, heart failure, diabetic nephropathy, and others) and conversion factors of different indications differed, this fact was indicated. In the final step the remaining drugs, not handled in previous steps, were categorised as not suitable for automatic switching thus requiring manual checking by a physician or pharmacist for possible alternatives, discontinuation, or external ordering of the brand.

**Table 1:** Drug classes integrated into the final CDSS version for automatic switching to therapeutic equivalents

**Drug class**	**ATC code(s)**
Antacids	A02AA, A02AB, A02AC, A02AD, A02AF, A02AH
Histamine H_2_-receptor antagonists	A02BA
Proton pump inhibitors	A02BC
Serotonin (5-HT_3_) antagonists	A04AA
Blood glucose lowering drugs, excl. insulins	A10BB, A10BG
Minerals	A12AA, A12BA, A12CB, A12CC
Antianemic preparations	B03AA
Thiazide diuretics	C03AA
Sulfonamides, plain (low-ceiling diuretics)	C03BA
Sulfonamides, plain (high-ceiling diuretics)	C03CA
Beta-blocking agents	C07AA, C07AB, C07AG
Calcium-channel blocking agents	C08CA
Angiotensin-converting enzyme inhibitors	C09AA
Angiotensin receptor antagonists	C09CA
HMG-CoA reductase inhibitors	C10AA
Fibrates	C10AB
Alpha-adrenoceptor antagonists	G04CA
Selective serotonin (5-HT_1_) agonists	N02CC
Benzodiazepine (anxiolytics)	N05BA
Benzodiazepine (hypnotics and sedatives)	N05CD
Benzodiazepine related drugs	N05CF

ATC: Anatomic Therapeutic Chemical classification system.

**Figure 3:**
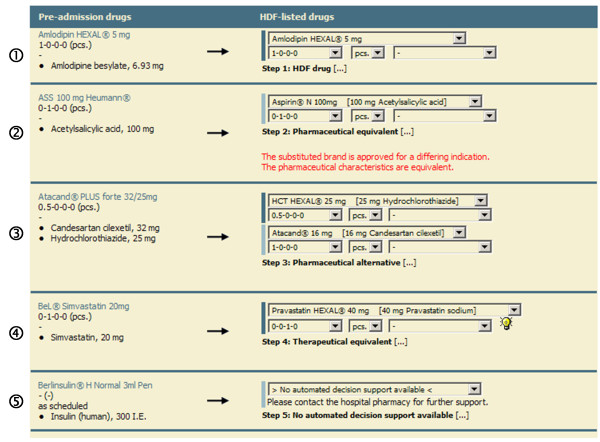
**Screenshot: CDSS for automatic switching of drugs.** Drugs prescribed to the patient before admission are listed on the left and the correspondingly switched drugs are shown on the right. The figure shows five examples of drug switches originating from the single steps of the algorithm (step 1–5). In example 2 an additional warning is displayed informing about differing indications of the switched drugs, in example 3 a combination drug is switched to two single agents. In example 4, the switch to a therapeutic equivalent required dosage adjustment (20mg simvastatin qd → 40mg pravastatin qd), and in example 5, no alternative could be suggested.

Based on this algorithm we implemented a web-based CDSS and integrated it into the existing CPOE (AiD*Klinik®*) used at the University Hospital of Heidelberg. For technical development we used PHP (PHP: Hypertext Preprocessor) and AJAX (Asynchronous JavaScript and XML) for the user interface (Figure 3), a MSSQL database management system (Microsoft SQL Server 2005) for data storage, and an IIS Webserver (Microsoft Internet Information Services 6.0) for providing the system to all 5,500 clients within the hospital. The required pharmaceutical and pharmacological knowledge was entered into the CDSS database using Microsoft Access 2003 data entry forms.

To implement the described algorithm, well structured data of all available drugs of the German market were necessary. Based upon this data (MMI Pharmindex, Medizinische Medien Informations GmbH, Germany) the CDSS compares different brands regarding important drug characteristics such as active ingredients, strengths, dosage forms, and ATC classification and also switches combination products. Depending on the step of the algorithm further subroutines of the CDSS provide additional data to be displayed (e.g. newly adjusted dosage regimens, information about tablet splitting, or hints and warnings related to the suggested substitution (Figure 3)).

### Evaluation of the CDSS

In some surgical wards of the University Hospital of Heidelberg switching drugs on admission is routinely performed by a team of clinical pharmacists. Therefore the patient’s drug history is documented by nurses or physicians of the surgical ward and faxed to the hospital pharmacy where the drugs are switched manually to drugs of the HDF. The resulting suggestion for in-house medication is faxed back to the requesting ward and documented on paper. These previously and independently documented medication switches were used to test the functionality of the newly developed CDSS (version 0.9). The medications of consecutively documented drug switch consultations of a three-month period were entered into the CPOE and the manual switches by the clinical pharmacists were compared with the suggestions of the electronic CDSS.

Because some drugs may be switched to more than one compound of the HDF each switch was evaluated by an independent senior clinical pharmacist who was blinded for the origin of the switch. The main goal of this comparison was to decide whether the switching results were identical, equivalent or whether either one suggestion was better. All switching results of the CDSS that were considered worse were reviewed once again by the same clinical pharmacist who judged whether the CDSS suggestion was inadequate/wrong or correct but suboptimal.

On the basis of this assessment the CDSS was slightly modified to further improve the software (version 1.0). Then the evaluation was repeated in an independent set of consecutive switch consultations documented in the three months following the first evaluation period.

### Data collection and analysis

This study was approved by the responsible Ethics Committee of the Medical Faculty of Heidelberg University, Germany (protocol # 136/2005) and conducted according the principles of the current version of the Declaration of Helsinki. Only anonymised prescription data of switch consultations were used for this study. Data were described with descriptive statistics and reported as absolute and relative frequencies or arithmetic means with standard deviation (SD). Data entry and analysis were performed on a Microsoft SQL Server 2005 database by using structured query language (SQL) and by using Microsoft Excel 2003.

## Results

### Pilot evaluation of the CDSS (version 0.9)

In the first evaluation 174 documented drug switch consultations were included containing 1,296 drugs manually switched by the team of clinical pharmacists (mean ± SD: 7.5 ± 4.3 drugs per consultation). 1,176 of these (90.7%) could be entered into the evaluation-database; the remaining 120 (9.3%) were excluded because essential information was missing on the handwritten consultations (e.g. drug strength or dosage regimen).

Of these 1,176 drugs 807 (68.6%) were substituted similarly by clinical pharmacists and the CDSS, i.e. identical drugs and dosage regimens were suggested. In the remaining 369 cases (31.4%) a different drug or a different dosage regimen resulted from automatic and manual switching. The blinded review of these by an independent expert revealed that in 42.0% of these cases suggestions of pharmacists and CDSS were equivalent, in 27.1% the CDSS suggestions and in 30.9% the pharmacists’ suggestions were considered superior. The detailed analysis of the latter group (30.9%) revealed that 78.1% of these CDSS suggestions were correct yet inferior to the pharmacists’ recommendations and 21.9% of the suggestions were inadequate or wrong, prompting modification of the first CDSS version. Hence, of the 1,176 switches that could be assessed 25 (2.1%) required modification. Weaknesses of version 0.9 of the CDSS mainly concerned four drug classes which were consequently excluded from automatic substitution:

1) “Insulins” (ATC code: A10A): Substitution of insulins should be personalised with a tailored monitoring of blood-glucose levels.

2) “Alpha-adrenoceptor blockers” (ATC code: C02CA): To switch alpha-adrenoceptor blockers a change of the dosage form and thus release characteristics (e.g. slow → instant release) can be necessary which is not yet supported by the given algorithm.

3) “Drugs for treatment of hyperkalemia and hyperphosphatemia” (ATC code: V03AE): In this group various agents with differing mechanisms of action are clustered that require manual processing (e.g. calcium carbonate → calcium diacetate).

4) “Other antianemic preparations” (ATC code: B03XA): In this group diverse chemical classes (e.g. biosimilars) are clustered that require processing by experts.

In the revised tool only oral dosage forms were considered thus omitting parenteral and inhaled drugs.

### Evaluation of the final CDSS (version 1.0)

The evaluation of the refined CDSS version comprised 202 documented drug switch consultations containing 1,518 drugs (7.5 ± 3.9 switches per consultation). Of these 185 prescriptions (12.2%) were incomplete or inaccurate and thus excluded leaving 1,333 drug switches (87.8%) for evaluation.

**Figure 4:**
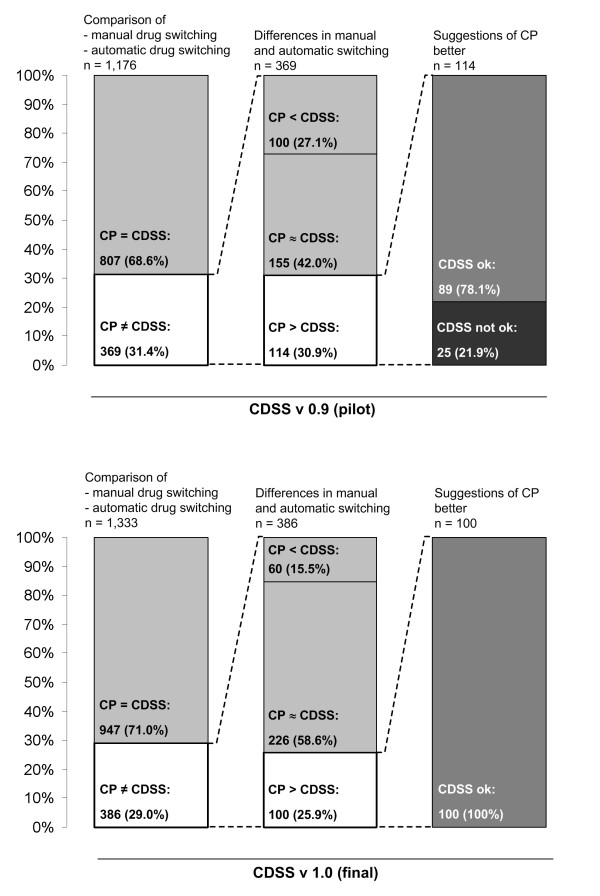
**Results of the evaluation.** Results of the pilot and final evaluation of the CDSS (computerised decision support system). The evaluation was performed by comparing the switch suggestions of the CDSS with those of the clinical pharmacists (CP). CP = CDSS: Drugs substituted similarly by CP and CDSS. CP ≠ CDSS: Relevant differences in automatic and manual switching. CP < CDSS: Suggestion of CDSS is better. CP ≈ CDSS: Both suggestions are considered appropriate. CP > CDSS: Suggestion of CP is better. CDSS ok: Suggestion of CDSS correct but inferior to suggestion of CP. CDSS not ok: Suggestion of CDSS inadequate/wrong.

947 of 1,333 drugs (71.0%) were substituted similarly by the team of pharmacists and the CDSS. Differences in automatic and manual switching occurred in 386 cases (29.0%). For 58.6% of these cases suggestions of pharmacists and CDSS were equivalent, in 15.5% the CDSS suggestions and in 25.9% the pharmacists’ suggestions were considered superior. The review of the latter discrepant suggestions revealed that all suggestions by the CDSS were correct, but not always the optimum choice, and in no instance (0%) inadequate equivalents were suggested. The results of both evaluations (version 0.9 and 1.0) are summarised in Figure 4.

### Switching performance of the CDSS

Ultimately the final version (version 1.0) was able to correctly suggest drugs of the HDF in 91.6% of all cases. In 31.7% the initial drug was identical with a drug of the HDF. In 40.7% an appropriate alternative with identical drug parent and strength was identified, and in 12.2% an alternative with identical parent but differing strength was found. In 7% a substitute with a different parent of the same drug class was suggested and equivalent doses were calculated. Only in 8.4% of all cases no substitute was found automatically thus requiring manual handling by an expert (Table 2). These latter drugs mainly belonged to the excluded drug classes, contained combination products with multiple active ingredients (>3), or did not meet other criteria necessary for automatic substitution (i.e. only solid oral dosage forms were switched to therapeutic equivalents ignoring e.g. inhaled compounds). Finally in some cases no possible substitutes were available and these brands had to be ordered from external suppliers.

**Table 2:** Performance of the CDSS

	**CDSS version 0.9 (n=1,176)**	**CDSS version 1.0 (n=1,333)**
Group 1: HDF drug	26.1%	31.7%
Group 2: Pharmaceutical equivalent	45.2%	40.7%
Group 3: Pharmaceutical alternative	14.0%	12.2%
Group 4: Therapeutic equivalent	8.3%	7.0%
Σ: CDSS-switch	93.6%*	91.6%
Group 5: No CDSS-switch	6.5%*	8.4%

*>100.0% due to rounding.

CDSS: Clinical decision support system, HDF: Hospital drug formulary.

## Discussion

In Germany close to 18 million people are hospitalised every year [12] and according to their drug history they are prescribed an average of six drugs [1,13]. Hence, every day roughly 300,000 drug switches are performed in German hospitals and – if performed with similarly poor accuracy as in our pilot study – they will be a major cause for avoidable risks for patients and also a waste of working force.

The potential for medication errors concerning dose adjustments after switching to therapeutic equivalents is well known. In an American study a significant proportion of patients whose cholesterol lowering medication was switched from atorvastatin to simvastatin thereafter received lower therapeutic doses, potentially impairing the quality of care and effectiveness [14]. But in some cases also the appropriateness of generic substitution is still controversially discussed [15,16]. Even when therapeutic doses and conversion factors are carefully considered the substitution may lead to critical changes in the exposure with additives [17,18] and – given the generally accepted range of bioequivalence – switching may cause considerably differing exposures to the active compound, which may be relevant for drugs with a narrow therapeutic window [15]. Therefore tight regulations and recommendations defining suitable drugs and drug classes for substitution might improve physicians’ confidence and compliance in the switching procedure [15].

Accordingly, we formed an interdisciplinary team of specialists (physicians, pharmacists, and computer scientists) to design and develop an electronic tool, which is a standard procedure to create well-fitted and user-friendly systems [19]. After implementation we evaluated a large independent sample of prospectively documented drug switches performed by experienced clinical pharmacists and thus used real clinical data as the most realistic test-cases.

The first test of our newly developed CDSS already showed good performance of our algorithm (93.6% could be switched electronically) but also revealed weaknesses that might have led to medication errors if the CDSS would have been released into clinical routine before meticulous validation. These weaknesses mainly albeit not exclusively concerned the switch to therapeutic equivalents, which in some cases required additional patient information or a switch to formulations with differing release characteristics. After modification, the second version (version 1.0) of the CDSS enabled automatic switching of 91.6% of the cases without any inadequate suggestions. Considering the huge size of the German drug market the performance of the tool is rather remarkable. Indeed with more than 70,000 pharmaceuticals, Germany has one of the largest drug markets worldwide suggesting that the CDSS will likely also efficiently switch drugs prescribed in other countries. Such a CDSS may even be useful in countries whose reimbursement system currently allows continuation of the patient’s own drugs during hospitalised care (such as the UK) because even then formulary substitutions are still required for example when patients are admitted as emergencies or if there is insufficient quantity of medicine to cover the whole inpatient stay.

Even in the optimised version, some drug switches suggested by the CDSS (100/1,333 observed switches) were judged inferior to the switching result of the clinical pharmacists. Analysis of these situations revealed that in most cases the clinical pharmacists derogated from the basic algorithm to improvise in a non-standard situation. For example our CDSS failed to compute an adequate dosage regimen after switching “Metoprolol 100 retard 1A Pharma” (containing 78.09 mg metoprolol) to “Beloc-Zok Retardtabl” (containing 77.82 mg metoprolol) because of slightly differing drug strengths, whose clinical irrelevance is easily recognised by an expert but requires proper specification for consideration by a computer system. Furthermore the human specialist is able to consult information sources beyond the CDSS database (e.g. by contacting the pharmaceutical manufacturer when additional drug information is needed and not available electronically). At last and in contrast to a CDSS, clinical pharmacists were able to consider special patient characteristics (e.g. age/mental state) and therefore to adjust a dosage regimen seeking to simplify the prescription (e.g. by avoiding tablet splitting). Indeed complex and complicated drug regimens (e.g. regimens with multiple administration times or the need for tablet-splitting) are an important prescription characteristic promoting non-adherence of the patients [20,21] that could be prevented in a large fraction of all prescriptions [22].

This emphasises areas of unmet need among professionals for support of drug switching in complicated cases that are not yet covered by the CDSS. Nevertheless, already today the CDSS can reduce the workload of these professionals by reliable handling of the large majority of routine substitutions. This is a substantial reduction of time when considering that manual drug switching by American clinicians was estimated to take 11 minutes per medication [23].

The thorough analysis of drug pairs not yet automatically switched revealed that a meaningful next step would be to support dose adjustment of different application forms and to consider combination products for therapeutic substitution (step 4). In addition, a future switching tool could also enable adoption of new scenarios like patient admission to an intensive care unit where oral forms often have to be switched to parenteral or intravenous forms and the switch back to ambulatory medication at discharge from hospital.

### Limitations

A number of potential limitations should be considered before generalisation of the results to other settings. (1) In our study we only switched drug combinations of surgical patients thus restricting evidence to patients receiving comparable medication regimens. However, as shown in our previous evaluation performed in the same wards [1], the Charlson score of these patients is high, reflecting the numerous co-morbidities of these patients and suggesting that their drug regimens likely represent also a population of internal medicine patients. (2) In our evaluation we had to exclude about 10% of the switch requests because essential details of the patients’ prescription were missing (e.g. missing dosage regimen, strengths, or information needed to identify the specific brand). This stresses the advantages of an electronic documentation of patient medications in a CPOE linked to drug databases as it enables exact identification of brands, the key information to relevant drug-related data like drug composition, strength, galenic formulation, and the corresponding SPC [24]. Unfortunately SPC information is currently not available in a well structured format [25], which would facilitate the development of tools like the one described herein. (3) Our evaluation was conducted by project members and not by clinical staff for whom the CDSS was developed. Thus, possible socio-technical incidents, a group of medication errors originating from interactions between clinical staff and the system [26], have yet to be investigated. (4) Finally the revision of differences between the switching results of pharmacists and the CDSS was performed by only one expert. This senior clinical pharmacist was considered best choice due to her extensive practical experience in switching drugs for years. However, by blinding this expert for the origin of the switching suggestions (pharmacist or CDSS) bias is minimised.

## Conclusions

The results of our study demonstrate that in the overwhelming majority of cases (>90%) a sophisticated electronic CDSS can safely and reliably switch drugs of admitted patients to the locally available drugs as compiled in a HDF. Given the substantial error-rates in this process such support is indeed needed.

## Abbreviations

HDF: Hospital drug formularies; CPOE: Computerised physician order entry; CDSS: Clinical decision support systems; SPC: Summary of product characteristics; ATC: Anatomic therapeutic chemical (classification system); SD: Standard deviation.

## Competing interests

The authors declare that they have no competing interests.

## Authors’ contributions

MGP participated in project coordination, conception of the study, system development, statistical analysis, and manuscript writing. SUWF participated in the development of the algorithm, analysis and interpretation of data, and manuscript writing. THT contributed to development of the algorithm, acquisition of data, and manuscript writing. JK supervised the system development and contributed to manuscript writing. WEH participated in project coordination, conception of the study, and manuscript writing. All authors read and approved the final manuscript.

## Pre-publication history

The pre-publication history for this paper can be accessed here:

http://www.biomedcentral.com/1472-6947/12/137/prepub
